# Supplementary material for: Noncontact human-machine interaction based on hand-responsive infrared structural color
Source: Nat Commun. 2022 Mar 18;13:1446. doi: 10.1038/s41467-022-29197-5 (PMC8933461; doi:10.1038/s41467-022-29197-5)
Supplement: Supplementary file 1 — Supplementary Information [file 41467_2022_29197_MOESM1_ESM.docx]

Supplementary Information

**Noncontact human-machine interaction based on hand-responsive infrared structural color**

Shun An^1^†, Hanrui Zhu^1^†, Chunzhi Guo^2^, Benwei Fu^1^, Chengyi Song^1^, Peng Tao^1^, Wen Shang^1^*, Tao Deng^1^*

^1^State Key Laboratory of Metal Matrix Composites, School of Materials Science and Engineering, Shanghai Jiao Tong University, 800 Dongchuan Road, Shanghai, 200240, China

^2^School of Electronic Information and Electrical Engineering, Shanghai Jiao Tong University, 800 Dongchuan Road, Shanghai, 200240, China

*Corresponding author. Email: [shangwen@sjtu.edu.cn](mailto:shangwen@sjtu.edu.cn) (W.S.); [dengtao@sjtu.edu.cn](mailto:dengtao@sjtu.edu.cn) (T.D.)

†These authors contributed equally to this work.

**This PDF file includes:**

Supplementary Figures 1 to 19

Supplementary Notes 1 to 6

Supplementary References

**Other Supplementary Information for this manuscript includes the following:**

Supplementary Movies 1 to 3


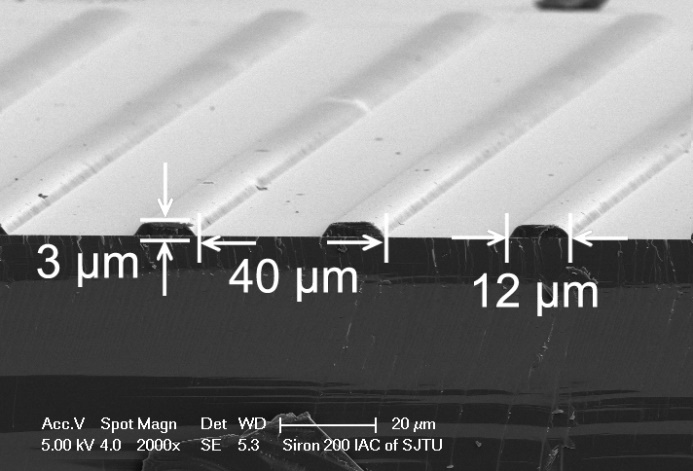


**Supplementary Figure 1. Cross-section SEM image of one grating fabricated in this study.** The SEM image shows the structural parameters of fabricated gratings.


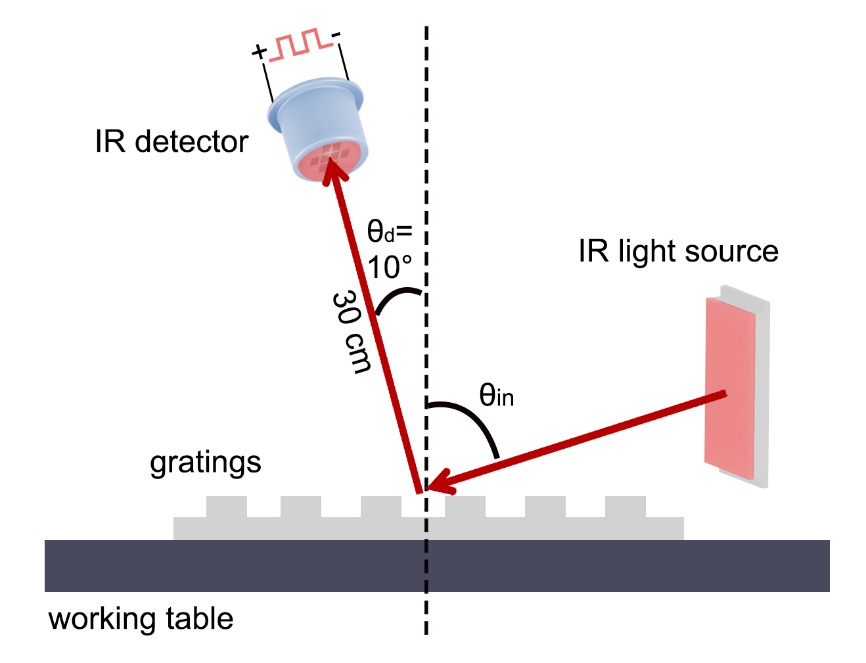


**Supplementary Figure 2. Experimental setup for the generation of IR structural colors.** The IR detector was placed about 30 cm away from the gratings. The lens of the IR detector was set at ~10° from the axis perpendicular to the center of the gratings.


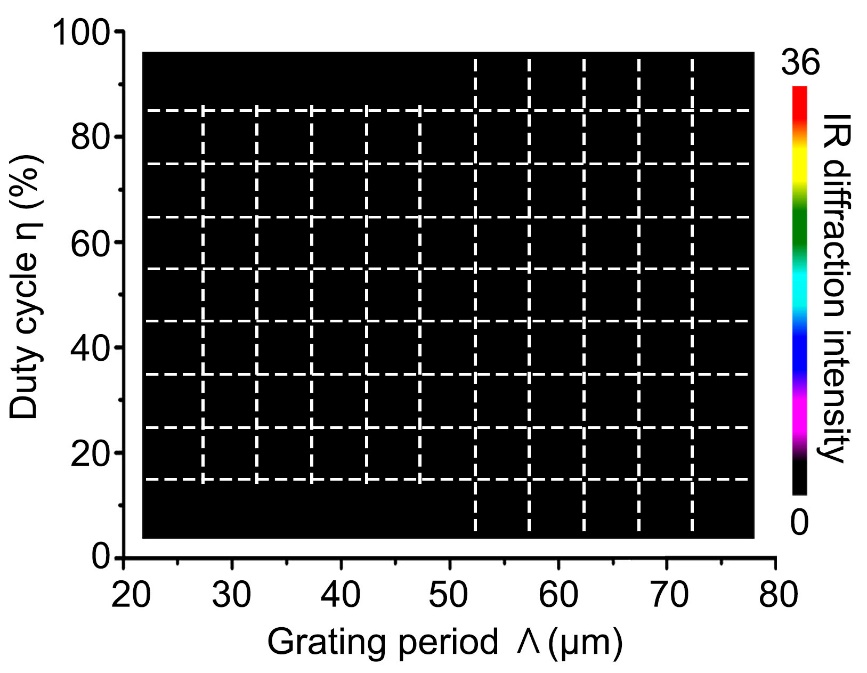


**Supplementary Figure 3. IR images of gratings without using the hand as the IR light source.** No color patterns were shown in the IR image of gratings with different grating periods and duty cycles without using the IR light source (hand).


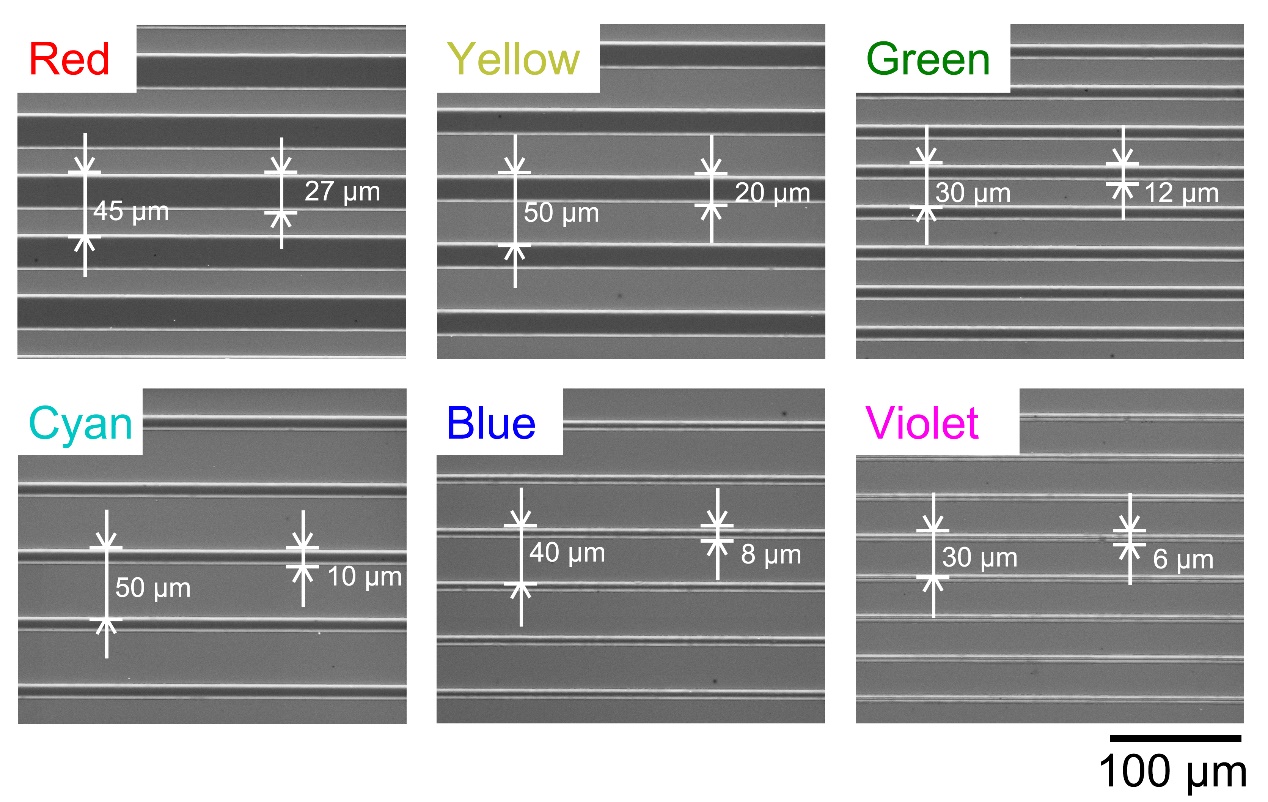


**Supplementary Figure 4. SEM images of gratings used for the generation of colorful “house”.** These SEM images show the structural parameters of fabricated gratings.


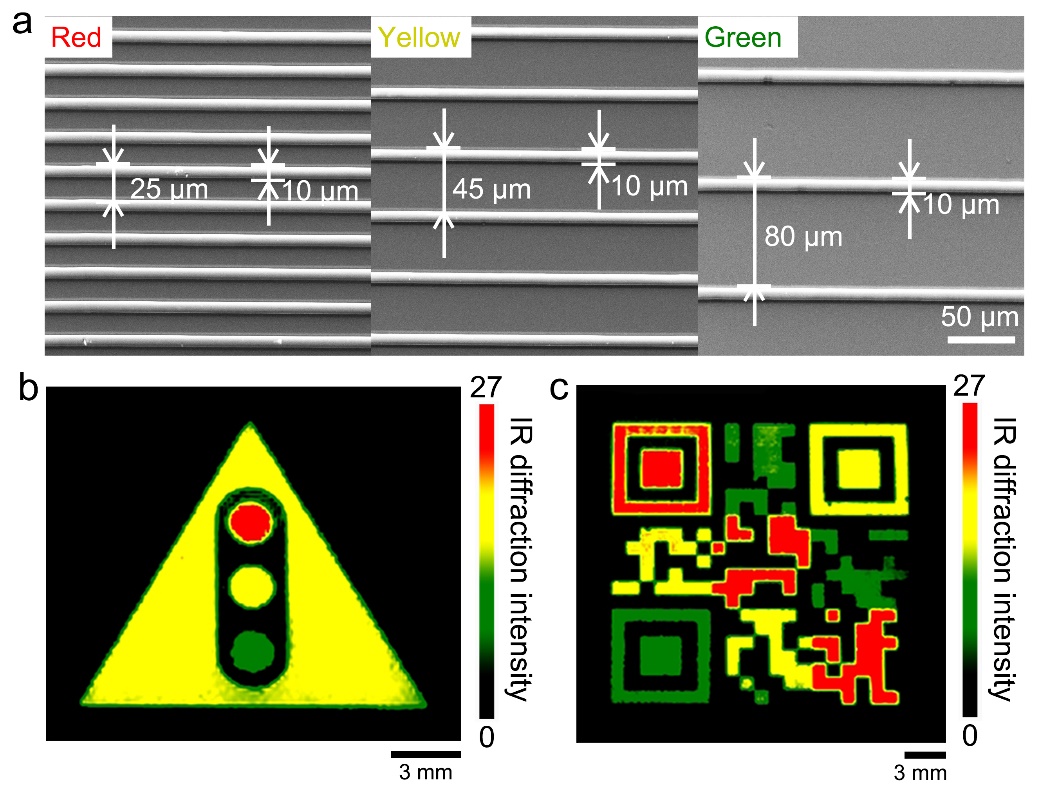


**Supplementary Figure 5. Applications of IR structural color. a** SEM images of three different gratings, which were used to generate different IR color patterns. **b** Color display of traffic lights. **c** 3D code generated for anti-counterfeiting.


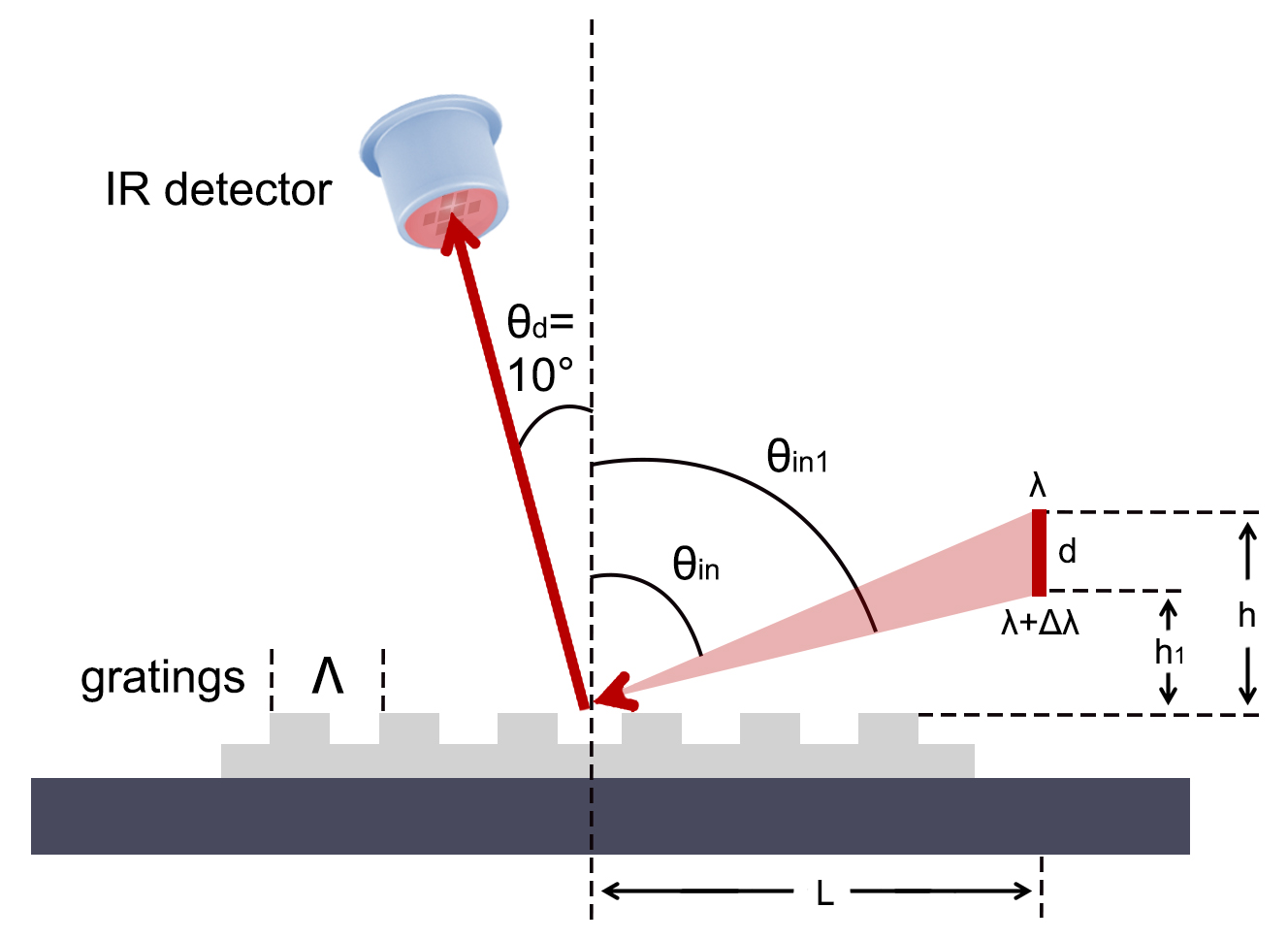


**Supplementary Figure 6. Experimental setup for the measurement of -1^st^ diffraction efficiency.** The incident angle (*θ_in_*) and the corresponding light wavelength (*λ*) are determined by the height (*h*) of the light source.


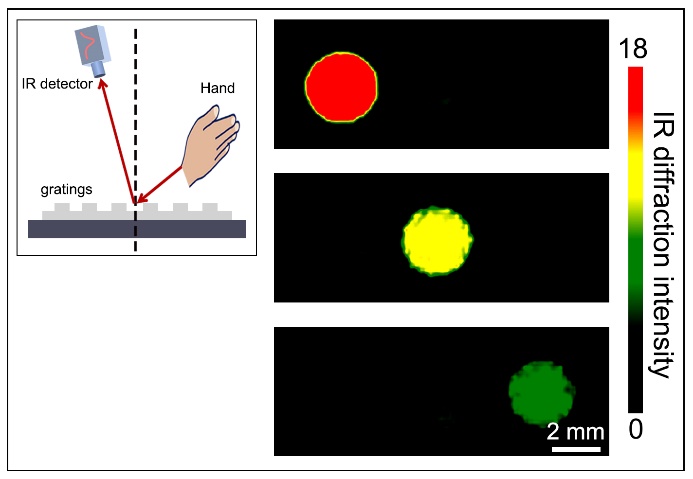


**Supplementary Figure 7. Dynamic generation of IR structural colors using the whole hand as the IR light source.** The red light, yellow light and green light are made of Gr, Gy, and Gg in Fig. 3e respectively. The inset shows the schematic of using the hand for the generation of IR structural colors.


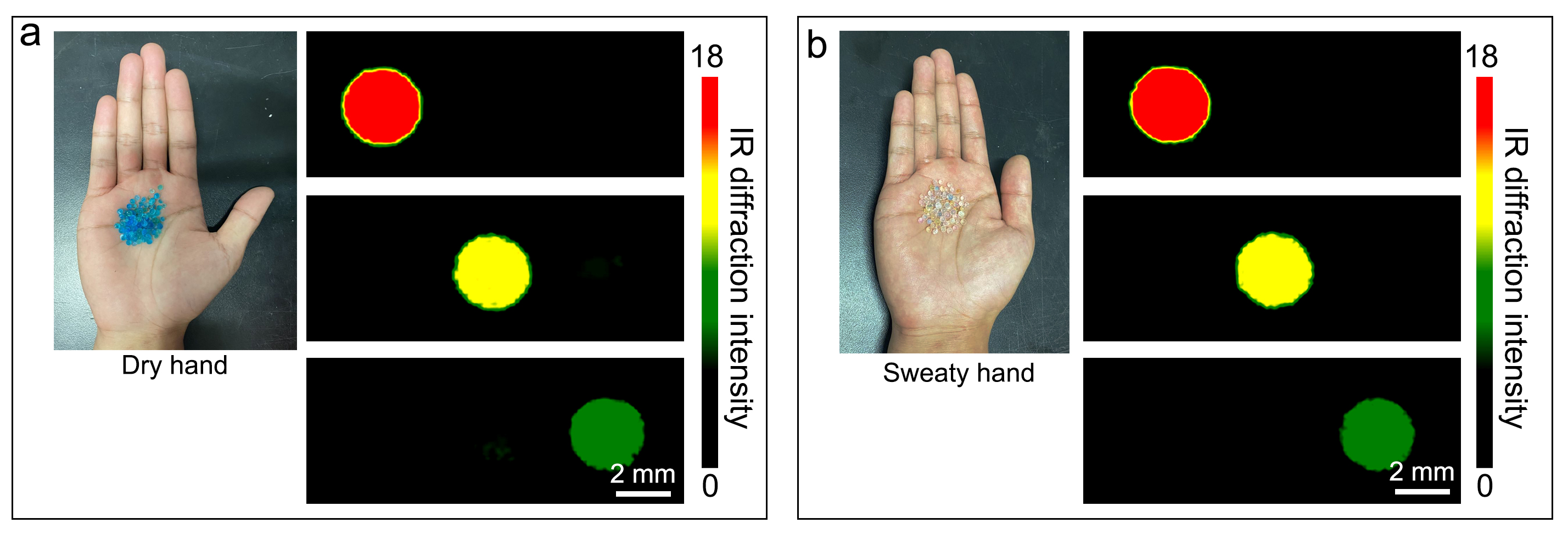


**Supplementary Figure 8. Effect of hand perspiration on the noncontact HMI. a** The generated IR color patterns using dry hand as the IR light source. **b** The generated IR color patterns using sweaty hand as the IR light source. The insets are the images of dry hand and sweaty hand. We used allochroic silica gel to characterize the perspiration of hand. The silica gel is blue under dry environment and will change from blue to relatively pink/translucent under humid environment due to the absorption of water vapor.


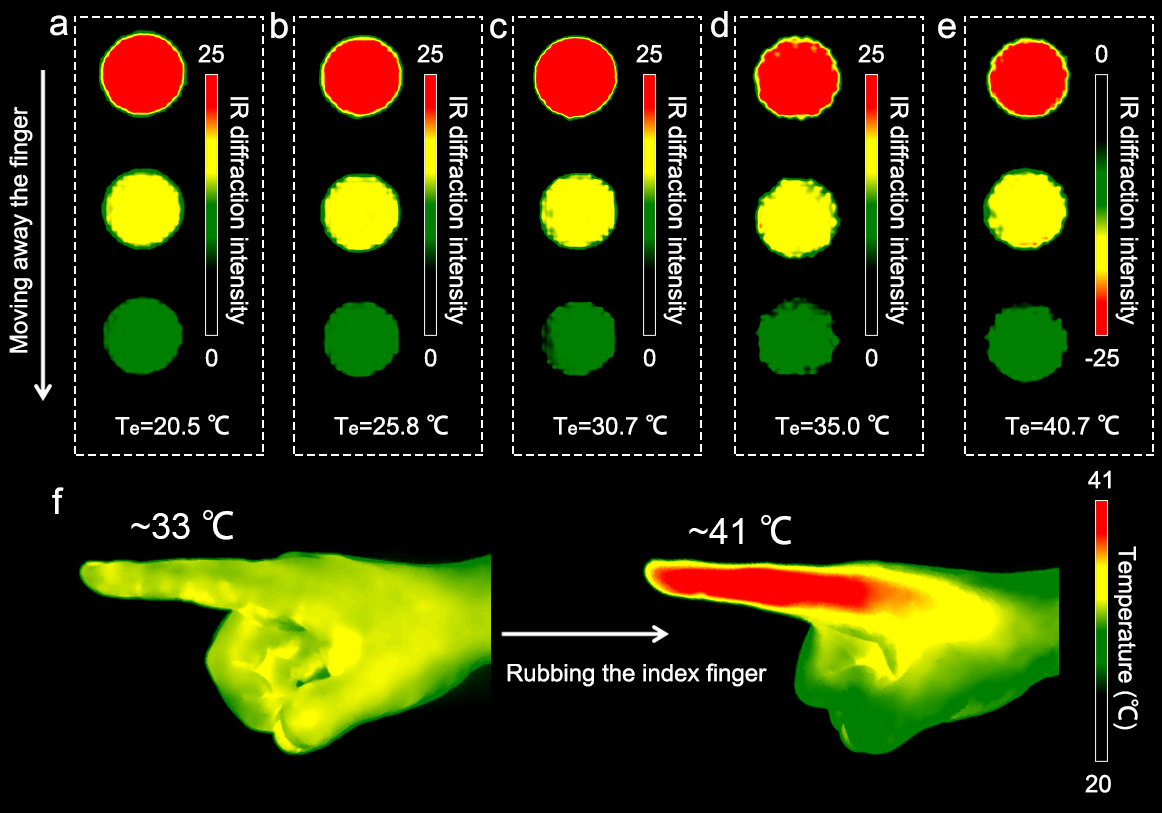


**Supplementary Figure 9. Effect of environmental temperature (T_e_) on the device performance. a-e** The generated IR structural colors under different environmental temperature. **f** IR images of the index finger before and after rubbing with the other hand.


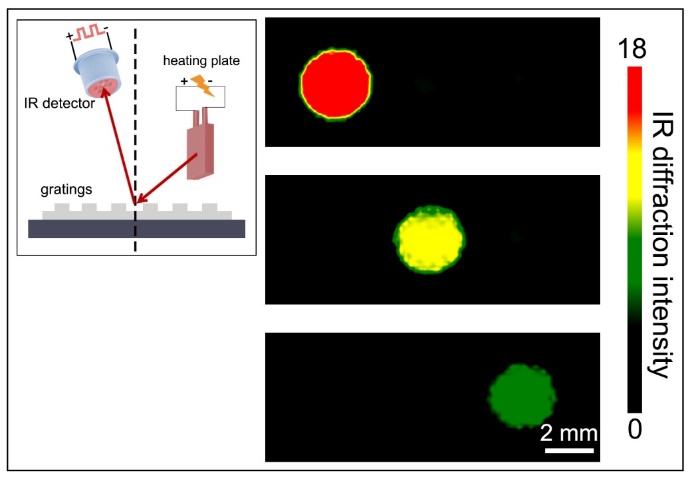


**Supplementary Figure 10. Dynamic generation of IR structural colors using a heating plate with the temperature of ~36 °C as the IR light source.** The inset shows the schematic of using the heating plate for the generation of IR structural colors.


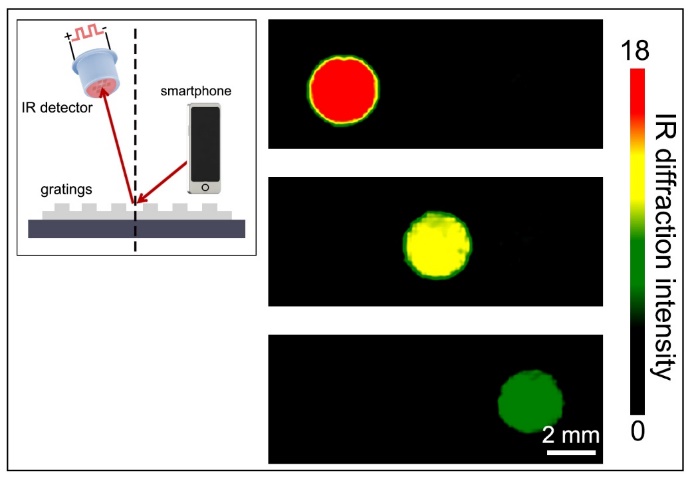


**Supplementary Figure 11. Dynamic generation of IR structural colors using a smartphone with the temperature of ~40 °C as the IR light source.** The inset shows the schematic of using the smartphone for the generation of IR structural colors.


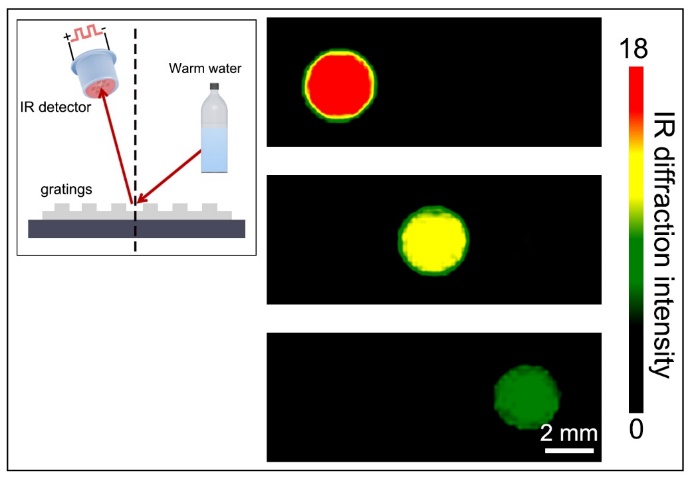


**Supplementary Figure 12. Dynamic generation of IR structural colors using a bottle of warm water with the temperature of ~45 °C as the IR light source.** The inset shows the schematic of using a bottle of warm water for the generation of IR structural colors.


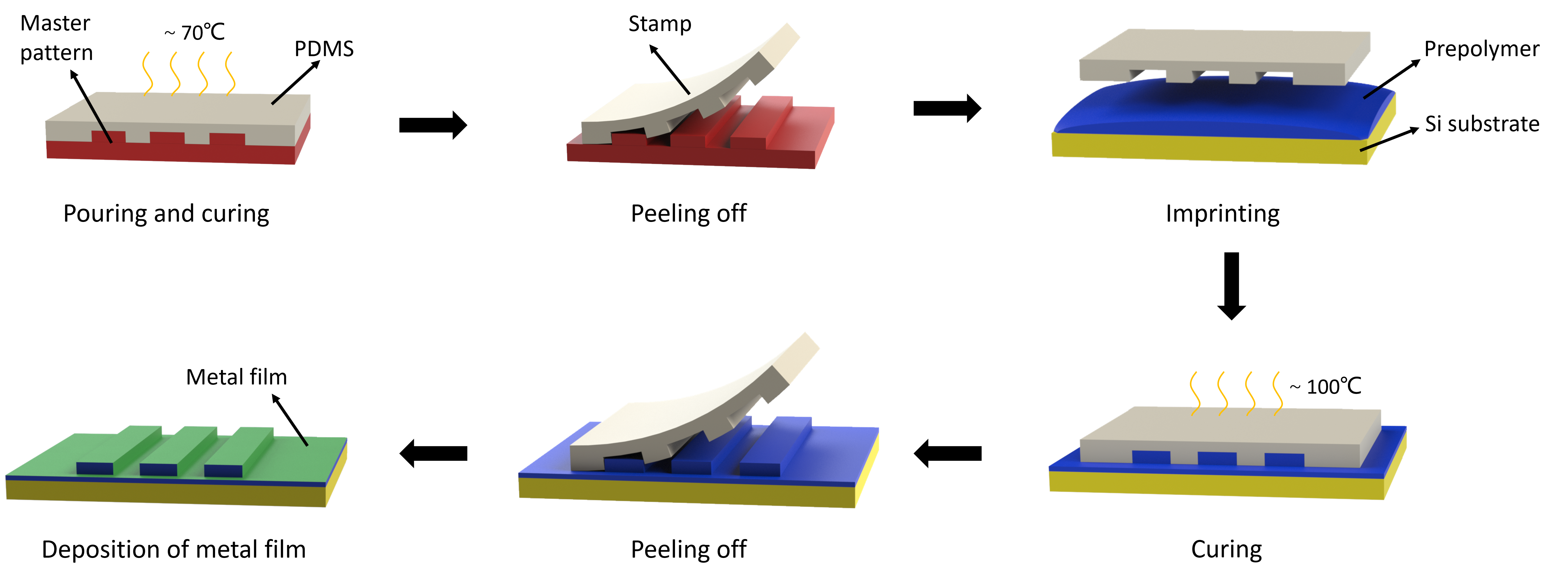


**Supplementary Figure 13. Schematic illustration of using soft lithography to fabricate grating arrays.** The schematic shows the fabrication process of grating arrays by soft lithography.


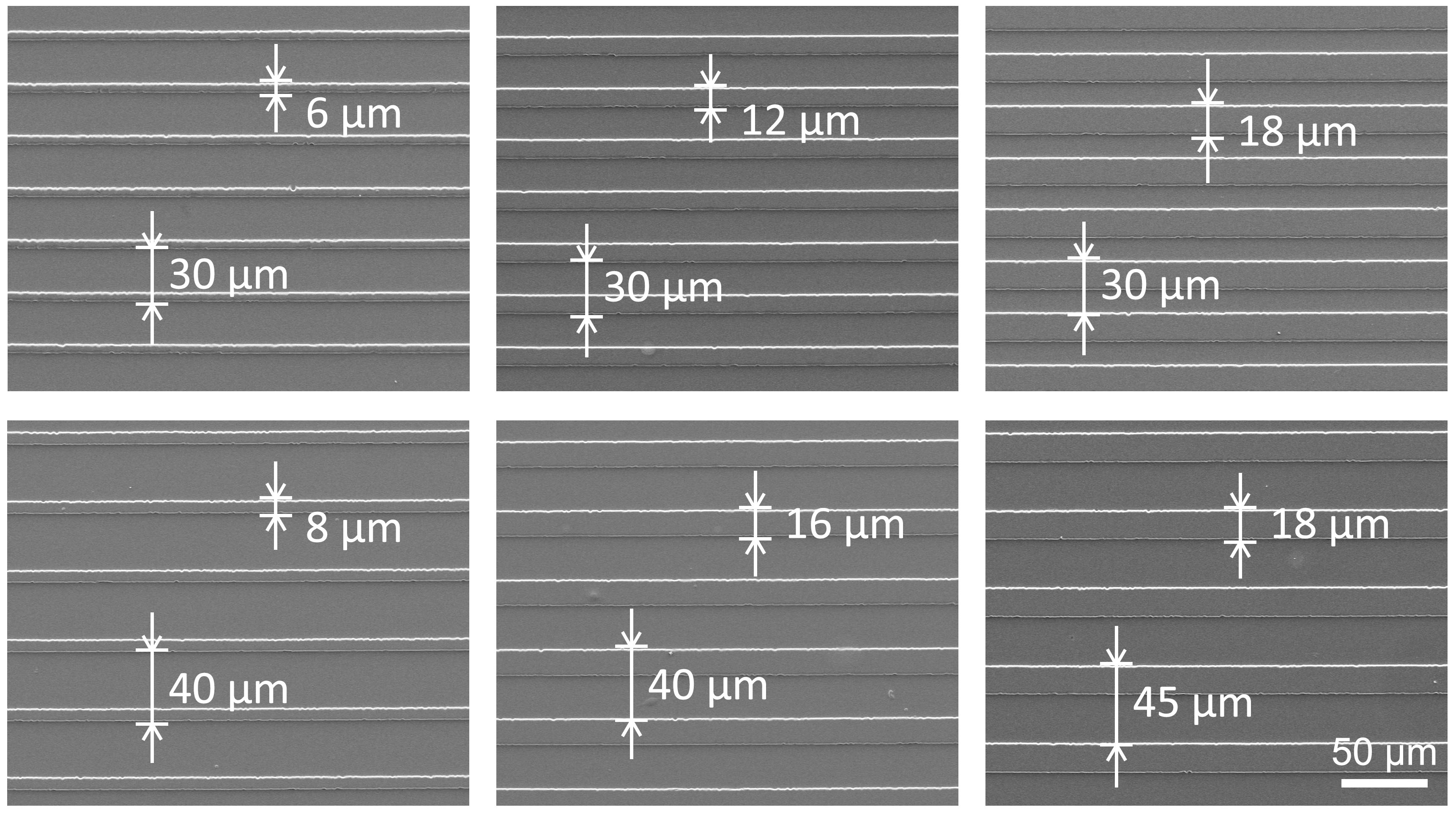


**Supplementary Figure 14. Fabrication of gratings on Si substrate by soft lithography.** These SEM images show the structural parameters of fabricated gratings.


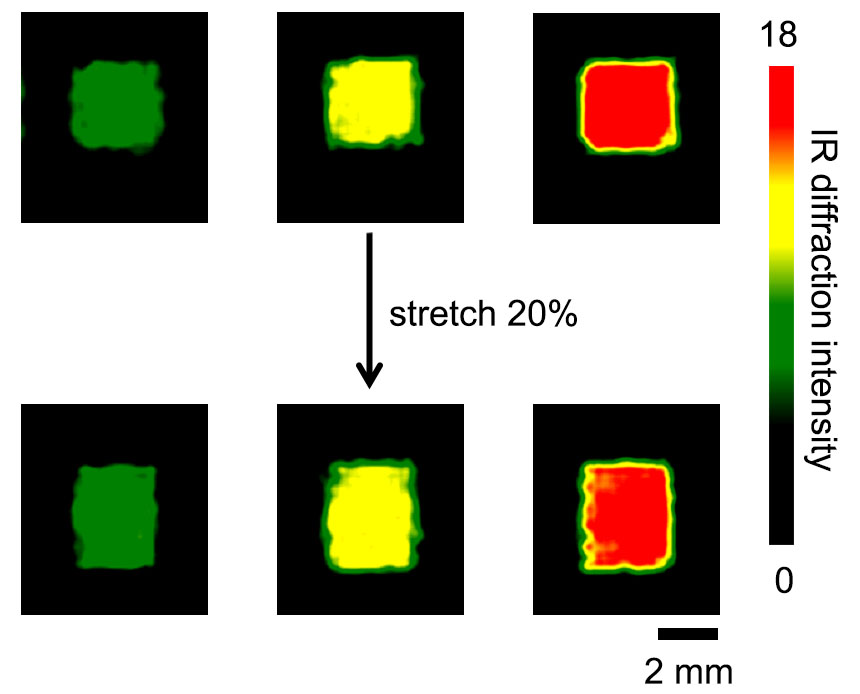


**Supplementary Figure 15.** **Fabrication of gratings on flexible PDMS substrate.** Top: The generated IR structural colors by placing the index finger in the incident angle range (20°~29°) of grating with the period of 45 μm. Bottom: The generated IR structural colors after the stretch of the sample by 20%. In this case, the period of the grating increased to 54 μm. The finger was placed in the corresponding incident angle range (18°~26°) to generate IR structural colors.


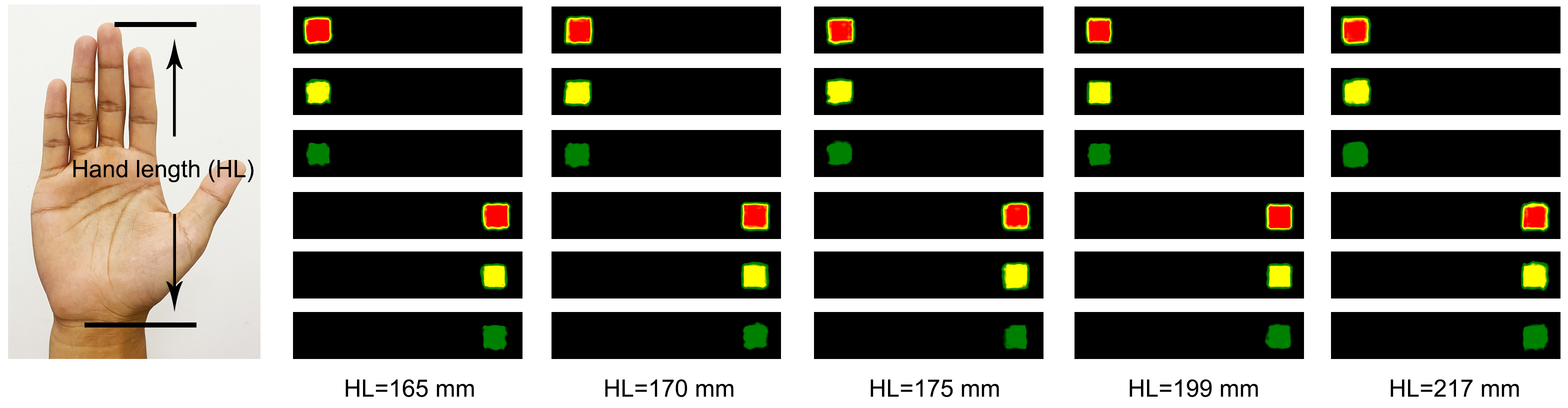


**Supplementary Figure 16. Generation of patterns of IR structural colors by operators with different hand length (HL).** The wrist band is applicable to operators with different hand length.


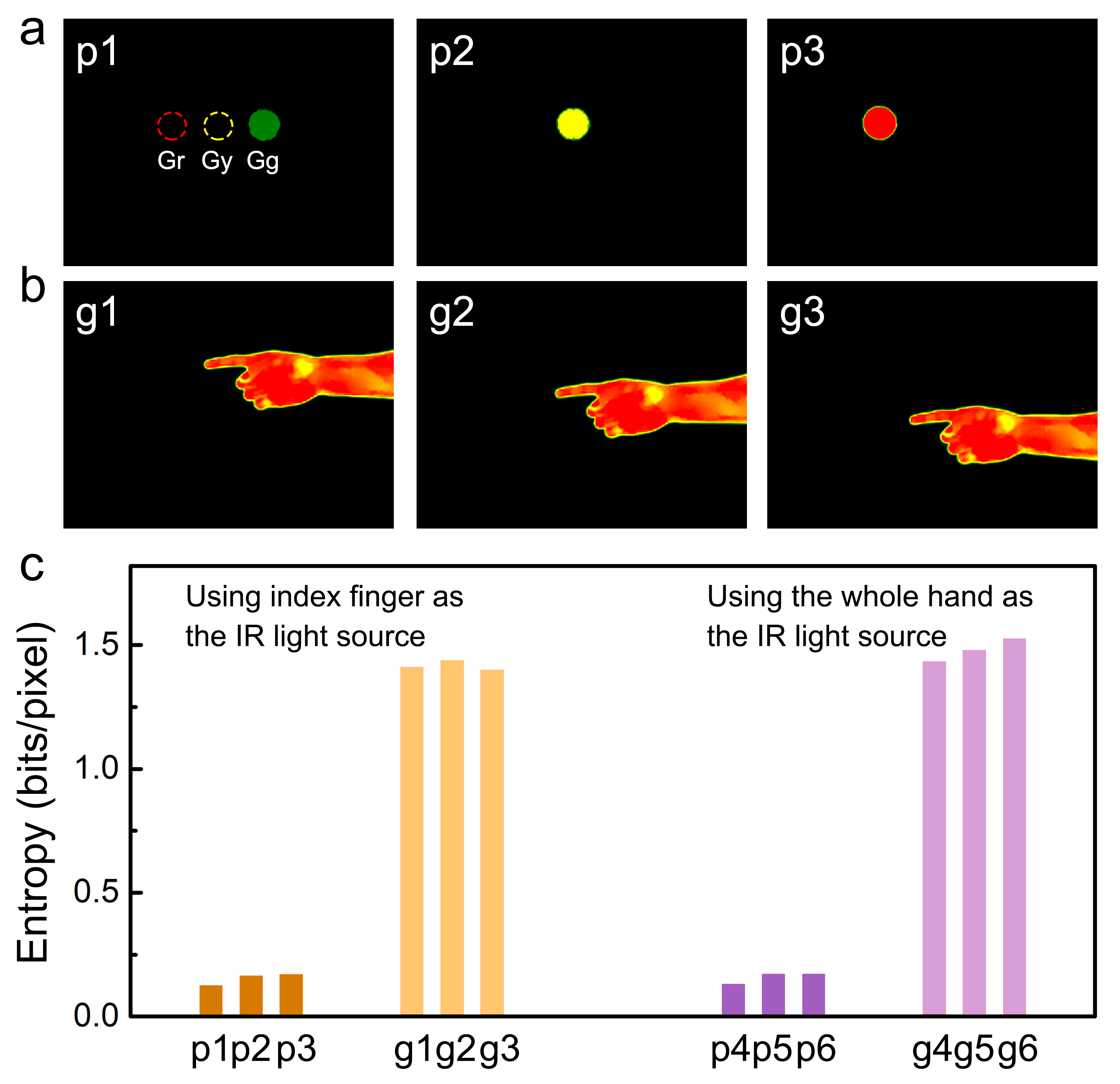


**Supplementary Figure 17. The comparison of information entropy of captured IR images. a** IR color patterns (p1, p2, and p3) generated by vertically moving the finger to the incident angle ranges of different gratings (Gg, Gy, and Gr). **b** Directly captured IR images of corresponding gestures (g1, g2, and g3) that generate the IR color patterns (p1, p2, and p3) in Supplementary Figure 17a. **c** The calculated entropy of the IR images in Supplementary Figure 17a and b (left two columns) and the calculated entropy of IR images by using the whole hand as the IR light source (right two columns). p4, p5, and p6 represent the generated IR color patterns by using the whole hand as the IR light source. g4, g5, and g6 represent the directly captured IR images of corresponding gestures that generate the IR color patterns (p4, p5, and p6).


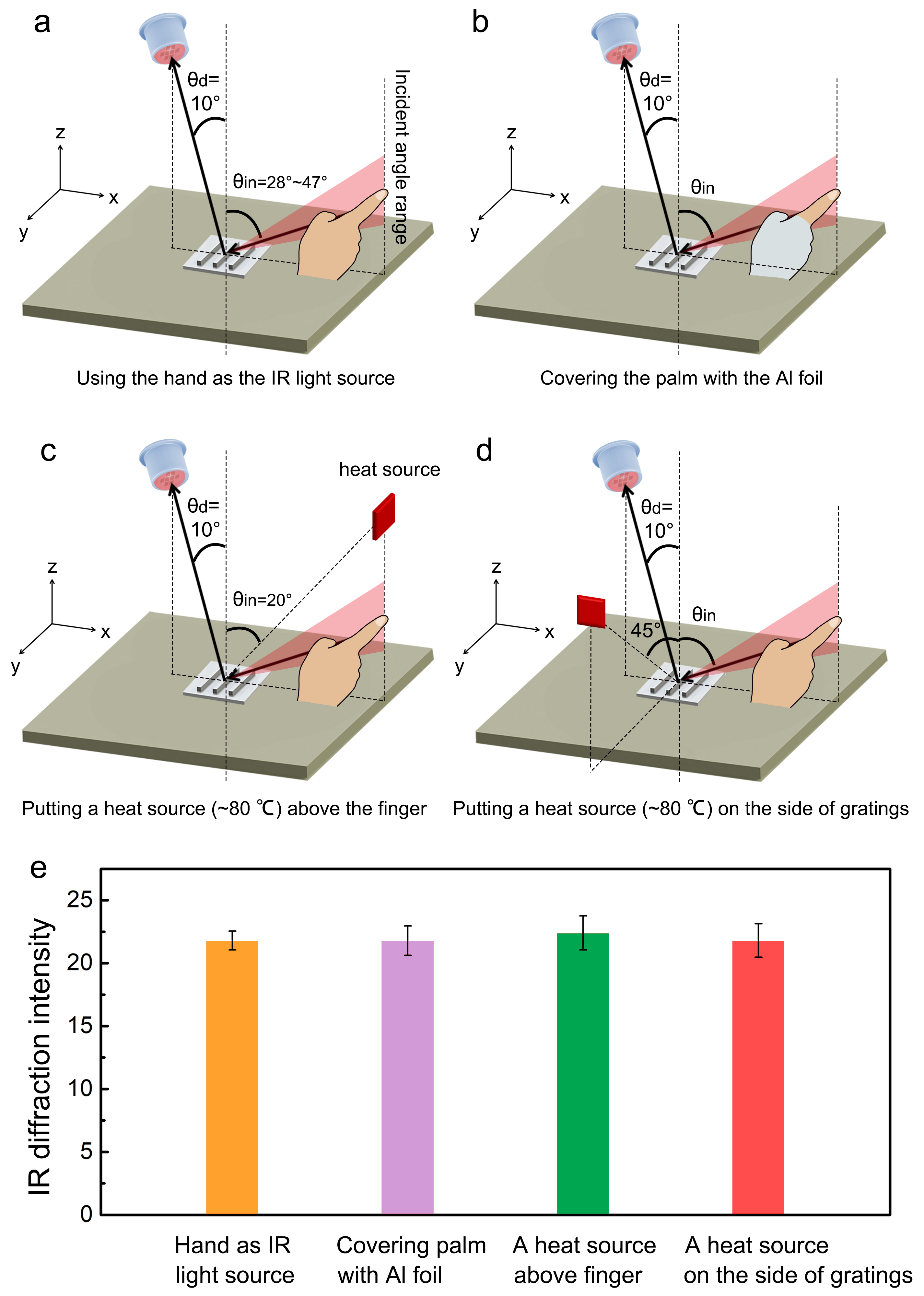


**Supplementary Figure 18. The reduction of interference from background and other parts of the hand due to the selective finger-grating interaction.** **a** Placing the index finger in the calculated incident angle range. **b** Covering the palm with the Al foil. **c** Placing a heat source with the temperature of ~80 ℃ above the finger. **d** Placing a heat source with the temperature of ~80 ℃ on the other side of gratings. **e** The IR diffraction intensity of the gratings under the above four conditions. Error bars represent the standard deviation of the mean.


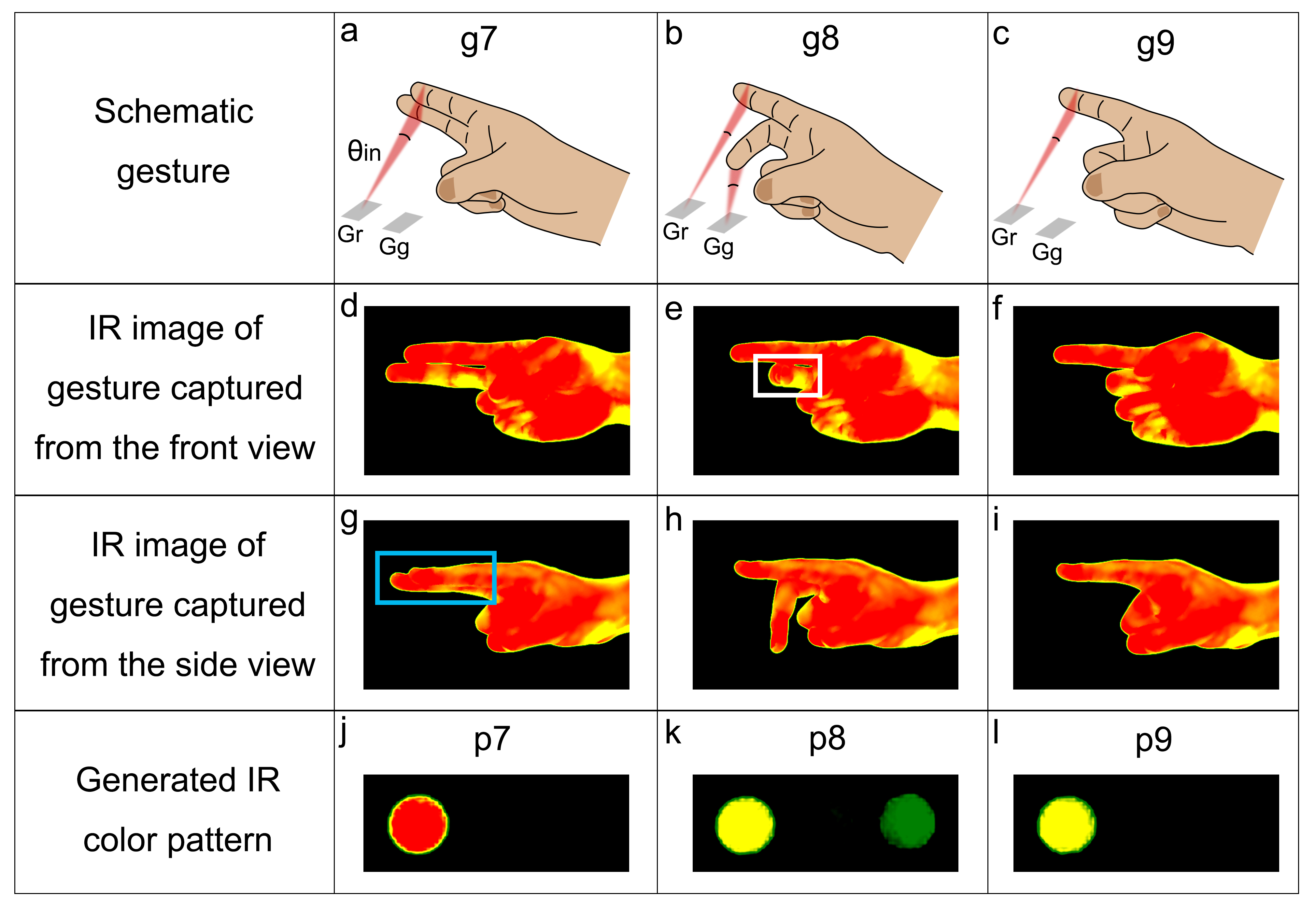


**Supplementary Figure 19.** **The characterization of the interference of self-occlusion on the recognition of features. a-c** Schematic illustration of three gestures (g7-g9). **d-f** Directly captured IR images of gestures from the front view. **g-i** Directly captured IR images of gestures from the side view. The white rectangle and the blue rectangle show the self-occlusion of gestures, which interferes the extraction of features. **j-l** Generated IR color patterns (p7-p9) by corresponding gestures.

**Supplementary Note**

**Supplementary Note 1. Mechanism of IR structural colors detected by the IR detector.**

In the IR imaging process, the IR emission, IR reflection, and IR diffraction are all collected by the IR detector as IR radiance. When there is no IR light source, the IR radiance from one grating is composed of the emitted IR radiation from the grating, the reflected IR radiation from the ambient, and the diffracted IR radiation from the ambient. As the ambient is in temperature equilibrium with the sample, the IR radiance from gratings with different periods or duty cycles has no difference and equals to the IR radiance from the ambient, which can be confirmed by the IR images of different gratings in Supplementary Figure 3. When there is an IR light source, for example, a human hand near the gratings, the temperature of hand is generally higher than that of the gratings or the ambient, which means the IR radiation intensity from hand is higher than that from environmental background. In this case, gratings will diffract IR radiation with increased intensity and the grating with higher diffraction efficiency diffracts IR radiation with higher intensity, which increases the detected IR radiance. When the ambient temperature is higher than hand temperature, the IR radiation intensity from hand is lower than that from environmental background. In this case, the IR diffraction intensity of the grating is decreased when the hand is used to interact with the grating, which decreases the detected IR radiance. The different IR radiance received by the IR detector is visualized as color distribution, that is, the IR structural color for different structures.

From the above analysis we can find that the different IR radiance from different gratings is derived from the different IR diffraction intensities. In the study, we used the normalized IR diffraction intensity to show the color differences in the images captured by the IR detector. In the normalized IR diffraction intensity, the IR radiance from gratings (equals to the IR radiance from the ambient) is set as 0 when no IR light source is used. The difference of IR radiance between the IR light source and the ambient is set as 100. Since the change of IR radiance is approximately proportional to the change of temperature, the normalized IR diffraction intensity (*I*) from one grating can thus be calculated by the equation:

$\text{I}=\frac{T_{grating}-T_{ambient}}{\text{|}T_{source}-T_{ambient}\text{|}}\cdot100$ (1)

where *T_grating_* is the apparent temperature reading of the grating, *T_ambient_* is the temperature reading of the ambient, *T_source_* is the temperature reading of the IR light source (human hand).

**Supplementary Note 2. FDTD calculation of the grating diffraction.**

The IR diffraction intensity was numerically calculated by the finite-difference time-domain (FDTD) method. The structural parameters (periods, duty cycles, thickness, etc.) of gratings were set according to the SEM images of gratings. The boundary condition of vertical direction was set as the perfectly matched layer (PML), which was based on the assumptions that the light was totally absorbed by the boundary in the vertical direction. The boundary condition of horizontal direction was set as periodic boundary condition (PBC) because the gratings were periodic in the horizontal direction. The material property of gratings was chosen as Al-CRC in database. The light source was set as the plane wave and the mesh size was set as 0.1 μm × 0.1 μm. The wavelength range was 7.5 μm to 14.0 μm, which is the detection range of the IR detector. For one specific wavelength (*λ*), the corresponding incident angle *θ_in_* was calculated according to the diffraction equation:^1^

$\text{sin}\theta_{\text{in}}=\sin\theta_{d}-\frac{m\lambda}{\Lambda}$ (2)

where *θ_d_* is the diffraction angle, which is 10° in the study. *m* is the diffraction order, which is nonzero integer. *λ* is the wavelength of the light. *Λ* is the grating period.

For one specific wavelength, the FDTD can calculate the corresponding diffraction efficiency, which can yield a curve of diffraction efficiency as a function of wavelength. The IR radiation intensity with specific wavelength satisfies the Planck distribution function:^2^

$M_{\lambda}(T)d\lambda=\frac{2\pi hc^{2}}{\lambda^{5}}\cdot\frac{1}{e^{hc/\lambda kT}-1}d\lambda$ (3)

Here, *h* is Planck’s constant, *c* is the speed of light in vacuum, *k* is Boltzmann constant, *λ* is the wavelength of the radiation, and *T* is the absolute temperature of the blackbody given in Kelvin. The IR diffraction intensity can be calculated by multiplying Equation (3) with the diffraction efficiency and then integrating the function from 7.5 μm to 14 μm. In this study, we normalized the IR diffraction intensity by setting the IR radiation intensity of the human hand with the temperature of 310 K as 100.

**Supplementary Note 3. Experimental measurement of the relationship between -1^st^ diffraction efficiency and light wavelength/incident angle.**

The diffraction efficiency (*ξ*) is defined as the ratio of IR diffraction intensity (*I_d_*) to the IR radiation intensity (*I_r_*):

$\xi=\frac{I_{d}}{I_{r}}$ (4)

In this work, when there is no hand or other IR light sources in the surroundings, the IR radiation from environmental background will interact with gratings and be diffracted. When hand or other IR light sources with temperature different from the environmental temperature is placed at specific position, the IR light source will replace the background and interact with gratings. Due to the IR radiation intensity of IR light source different from that of background, the IR diffraction intensity changes. The diffraction efficiency (*ξ*) can thus be calculated by:

$\xi=\frac{\Delta\text{I}_{d}}{\Delta I_{r}}$ (5)

where *ΔI_d_* is the change of IR diffraction intensity with the IR light source placed at specific position. *ΔI_r_* is the change of IR radiation intensity with the IR light source placed at specific position, compared with the IR radiation intensity of background.

According to diffraction equation (Equation 2), for the fixed diffraction angle (*θ_d_* = 10°) and specific grating period *Λ*, the incident angle (*θ_in_*) is relative to the light wavelength (*λ*). The detected wavelength range is 7.5 μm (*λ_1_*)~14 μm (*λ_2_*). Each wavelength corresponds to one specific incident angle.

To measure the relationship between -1^st^ diffraction efficiency (*ξ*) and light wavelength (*λ*)/incident angle (*θ_in_*), we used a heating rod with the diameter of *d* = 4 mm and the temperature of *T_h_* = 80 °C as an IR light source. The experimental setup is shown in Supplementary Figure 6. The horizontal distance between the grating and light source is *L* = 9 cm. The environmental temperature is *T_e_* = 24.3 °C.

When the IR light source is placed in the calculated incident angle range, the IR radiation is diffracted by the grating and then detected by the IR detector, which is characterized as the increase of the apparent temperature of grating from *T_e_* to *T_d_*. The increased IR diffraction intensity (*ΔI_d_*) can thus be calculated based on Planck distribution function (Equation 3):

$\Delta\text{I}_{d}=\int_{\lambda_{1}}^{\lambda_{2}} M_{\lambda}(T_{d})d\lambda-\int_{\lambda_{1}}^{\lambda_{2}} M_{\lambda}(T_{\text{e}})d\lambda$ (6)

Due to the small size of the IR light source, it can only cover a small range of incident angle, corresponding to a small range of light wavelength (*λ*, *λ+Δλ*). Compared with using the environmental background as the IR light source, the increase of IR radiation intensity (*ΔI_r_*) by using the heating rod as the IR light source (*T_h_* = 80 °C) can be calculated:

$\Delta\text{I}_{\text{r}}=\int_{\lambda}^{\lambda+\Delta\lambda} M_{\lambda}(T_{\text{h}})d\lambda-\int_{\lambda}^{\lambda+\Delta\lambda} M_{\lambda}(T_{\text{e}})d\lambda$ (7)

The detected wavelength range (*λ*, *λ+Δλ*) is related to the angle of the IR light source. First, we can calculate the incident angle range (*θ_in_*, *θ_in1_*):

$\tan\theta_{\text{in}}=\frac{L}{h}$ (8)

$\tan\theta_{\text{in1}}=\frac{L}{h_{1}}$ (9)

$h_{1}=h-d$ (10)

where *h* is the height of the light source.

The detected wavelength range (*λ*, *λ+Δλ*) is thus can be calculated by the diffraction equation (Equation 2):

$\lambda=\Lambda\cdot(\sin\theta_{in}-\sin\theta_{d})$ (11)

$\lambda+\Delta\lambda=\Lambda\cdot(\sin\theta_{in1}-\sin\theta_{d})$ (12)

Combining Equation 8-12, we can deduce the relationship between the detected wavelength range (*λ*, *λ+Δλ*) and the height of the light source:

$\lambda=\Lambda\cdot(\mathrm{sinarctan}\frac{L}{h}-\sin\theta_{d})$ (13)

$\lambda+\Delta\lambda=\Lambda\cdot(\mathrm{sinarctan}\frac{L}{h\text{-}d}-\sin\theta_{d})$ (14)

Combining Equation 13, 14, we can find that:

$\Delta\lambda=\Lambda\cdot(\mathrm{sinarctan}\frac{L}{h\text{-}d}-\mathrm{sinarctan}\frac{L}{h})$ (15)

Since *d*<<*L* and *h*, *Δλ* is thus a small value. We simplify the Equation 7 as:

$\begin{matrix} \Delta\text{I}_{\text{r}}=\int_{\lambda}^{\lambda+\Delta\lambda} M_{\lambda}(T_{\text{h}})d\lambda-\int_{\lambda}^{\lambda+\Delta\lambda} M_{\lambda}(T_{\text{e}})d\lambda=\Delta\lambda\cdot(M_{\lambda}(T_{\text{h}})-M_{\lambda}(T_{\text{e}})) \\ =\Lambda\cdot(\mathrm{sinarctan}\frac{L}{h\text{-}d}-\mathrm{sinarctan}\frac{L}{h})\cdot(M_{\lambda}(T_{\text{h}})-M_{\lambda}(T_{\text{e}})) \end{matrix}$ (16)

The relationship between diffraction efficiency (*ξ*) and light wavelength (*λ*)/incident angle (*θ_in_*) can thus be calculated by combing Equation 6 and Equation 16:

$\xi(\lambda)=\frac{\int_{\lambda_{1}}^{\lambda_{2}} M_{\lambda}(T_{d})d\lambda-\int_{\lambda_{1}}^{\lambda_{2}} M_{\lambda}(T_{\text{e}})d\lambda}{\Lambda\cdot(\mathrm{sinarctan}\frac{L}{h\text{-}d}-\mathrm{sinarctan}\frac{L}{h})\cdot(M_{\lambda}(T_{\text{h}})-M_{\lambda}(T_{\text{e}}))}$ (17)

In this experiment, we moved the IR light source in the incident angle ranges of three different gratings (*Λ* = 25 μm, 40 μm, and 60 μm respectively) and recorded the height (*h*) and the apparent temperature of the grating (*T_d_*). Combining Equation 17 and the data of *h* and *T_d_*, we can calculate the relationship between diffraction efficiency (*ξ*) and light wavelength (*λ*)/incident angle (*θ_in_*). To simplify the calculation process, we calculated the relative diffraction efficiency *ξ* (arbitrary units) of radiation with different wavelength.

**Supplementary Note 4. Human-machine interaction based on IR structural colors.**

In this proof-of-concept demonstration, we used the index finger of the hand as the IR light source to generate different patterns of IR structural colors based on three different gratings and used the generated color signals to command a robot vehicle to perform different actions. The task of the robot vehicle is to transport an object to a destination. The robot vehicle first needs to lift up the object and then move to the destination. At the destination, the car puts down the object. We used Matlab (MATLAB R2018b) to acquire the video stream of the generated IR structural colors in real time. For one frame of the video, the RGB values of the center pixel for each of the three gratings are read. Based on the RGB values, different commands are recognized. For example, if the RGB values are (255, 0, 0), (0, 0, 0), and (0, 0, 0) respectively, this frame of IR structural colors is recognized as “move forward”. If the index finger does not interact with any of the three gratings, this frame of IR structural colors is shown as all black and recognized as “stop”. The commands are sent to the Bluetooth of Arduino UNO integrated on the robot vehicle and the robot vehicle thus can perform different actions based on received commands.

**Supplementary Note 5. Calculation of two-dimensional (2D) information entropy of captured IR images**

2D information entropy is defined by the grayscale distribution of an image, which can characterize the amount of data in the image. For each pixel of the image, the grayscale of the pixel and the average grayscale of its neighborhood are first calculated, which are *i* and *j* respectively. The grayscale of the pixel (*i*) and the average grayscale of the neighborhood (*j*) form a pair. The probability of each pair is then calculated to be *p_ij_*. The 2D entropy can be calculated by the formula^3^:

$E=-\sum_{i=0}^{255} \sum_{j=0}^{255} p_{ij}\log_{2}p_{ij}$ (18)

**Supplementary Note 6. Comparison between direct gesture-based HMI and IR structural color-based HMI.**

IR camera is also widely used to directly capture gestures. The captured gestures are recognized as specific commands for HMI, which can be referred as direct gesture-based HMI. Compared with direct gesture-based HMI, the conversion of complex gestures into simplified IR color patterns can decrease the amount of information in the captured image and thus is beneficial for real-time recognition. The amount of information of an image can be characterized by information entropy (Supplementary Note 5)^4^. The smaller entropy means the less amount of information. In Fig. 3 we demonstrate that we can use individual finger as the IR light source to interact with the grating array and generate different patterns of IR structural colors by vertically moving the finger to the incident angle ranges of different gratings (Gr, Gy, and Gg). Here we quantitively compared the entropy of two processes (IR structural color-based imaging and direct IR imaging of gestures) by using one finger. As shown in Supplementary Figure 17a, we can generate three different IR color patterns (p1, p2, and p3) by moving the index finger in the IR structural color-based imaging process. To compare with the direct IR imaging of gestures, we also directly captured the IR images of the same gesture at the positions where these three IR color patterns were generated. The directly captured IR images of the gesture at different positions, which are named as g1, g2, g3, are shown in Supplementary Figure 17b. We calculated the information entropy of these IR images respectively. As shown in Supplementary Figure 17c (left), the information entropy of directly captured IR images of gestures (1.42 bits/pixel on average) is much larger than that of IR color patterns (0.16 bits/pixel on average). In Supplementary Figure 7, we also demonstrate that we can use the whole hand as the IR light source to interact with the grating array and generate different patterns of IR structural colors. Here we compared the entropy of two processes (IR structural color-based imaging and direct IR imaging of gestures) by using the whole hand as well. The comparison process is the same as the above comparison process, with the generated IR color patterns named as p4, p5, and p6 and the corresponding gestures named as g4, g5, and g6 respectively. As shown in Supplementary Figure 17c (right), the average information entropy of IR color patterns generated by the gesture of the whole hand is 0.16 bits/pixel, also much less than the average information entropy of gesture of the whole hand (1.48 bits/pixel). The smaller information entropy means that IR color patterns contain less amount of information, which is beneficial for real-time recognition.

In direct gesture-based HMI, the IR camera captures the IR image of the whole hand and the background. The complex background and the other parts of the hand will interfere the recognition of the gesture^5,6^. In comparison, this IR structural color-based HMI uses the grating array as the interface to selectively interact with fingers, which minimizes the interference of background and other parts of the hand. To demonstrate such minimized interference due to the selective interaction between hand and gratings, we used Gr with the period of 25 μm (Fig. 3e) to interact with the hand (Supplementary Figure 18). For the detected diffraction angle (*θ_d_* = 10°), the corresponding incident angle range is calculated to be 28°~47°. When we placed the index finger in the calculated incident angle range, the IR radiation from the finger can be diffracted by the grating with the diffraction angle of *θ_d_* = 10° (Supplementary Figure 18a). Apparently, IR radiation from the other parts of the hand (the palm) and the background can also interact with the grating. To confirm whether the IR radiation from other parts of the hand can interfere the generation of IR color patterns, we used an Al foil with low emissivity (~0.01) to cover the hand except the index finger, which limits the IR radiation from other parts of the hand (Supplementary Figure 18b). We compared the IR diffraction intensity of the grating with the hand not covered and covered with the Al foil. As shown in Supplementary Figure 18e, the cover of the hand with Al foil does not change the IR diffraction intensity of the grating, which means that the IR radiation from the other parts of the hand does not interfere the selective interaction between the index finger and the grating. We also placed a heat source with the temperature of ~80 ℃ at different positions (Supplementary Figure 18c and 18d) and compared the IR diffraction intensity of the grating before and after adding the heat source. As shown in Supplementary Figure 18e, the extra heat source does not change the IR diffraction intensity of the grating, which means that only the IR radiation with the calculated incident angle can selectively interact with gratings and detected by the IR camera. Other IR radiation does not interfere the generation of IR structural colors. The selective interaction between hand and gratings minimizes the interference of complex background and other parts of the hand on the recognition of gestures and commands.

Direct gesture-based HMI generally involves gestures composed of multiple fingers. In this case, self-occlusion is hard to avoid because the hand is a complex articulated object with more than 20 degrees of freedom^6,7^. The self-occlusion of gestures will interfere the extraction of features from gestures and thus affect the recognition performance of HMI. In the IR structural color-based process, the highly selective interaction between individual finger and the corresponding grating minimizes the impact of self-occlusion, since grating only interacts with the corresponding finger, while other fingers or other parts of the hand will not interfere such interaction. We further ran an experiment to use two fingers in the two processes (direct IR imaging of gestures and the IR structural color-based imaging) to compare the effect of self-occlusion on the generated IR images. This experiment involves three gestures (g7, g8, g9 in Supplementary Figure 19a-c) for the direct imaging process. For comparison, in the IR structural color-based process, we used two gratings (Gr and Gg in Fig. 3e) to interact with these three same gestures. As shown in Supplementary Figure 19a, we first used the extending middle finger and index finger as the gesture (g7). We then bent the middle finger to 90° to generate a new gesture (g8, Supplementary Figure 19b). We further bent the middle finger to the palm and generated another gesture (g9, Supplementary Figure 19c). For the first process that involves the direct capturing of the IR images of the three gestures, we captured the three gestures using IR camera from two different angles (front view and side view). From the directly captured IR images of gestures (Supplementary Figure 19d-i) we can find that self-occlusion is hard to avoid and interferes the extraction of gesture features. For example, in the IR image of g8 captured from the front view (Supplementary Figure 19e), the bending part of the middle finger is overlapped in this angle (white rectangle), which makes the feature of bending the middle finger unrecognizable in the captured IR image and makes the differentiation of IR images of g8 (Supplementary Figure 19e) and g9 (Supplementary Figure 19f) a little challenging. When the IR camera captured gestures from the side view, the bending of the middle finger is recognizable (Supplementary Figure 19h). In this case, however, it is hard to differentiate the captured IR image of g7 (Supplementary Figure 19g) from that of g9 (Supplementary Figure 19i) due to the block of the middle finger by the index finger (blue rectangle). The self-occlusion is thus hard to avoid in the direct gesture-based HMI, which interferes the extraction of features from gestures and the recognition performance of gestures. In comparison, for the IR structural color-based process, we captured the IR color patterns using the same three gestures. We first placed the gesture g7 in the incident angle range of Gr to generate the IR diffraction pattern of “red” on Gr (p7, Supplementary Figure 19j). We then bent the middle finger to 90° to generate a gesture g8. The bending part of the middle finger covered the incident angle range of Gg and generated the IR diffraction pattern of “green” on Gg (p8, Supplementary Figure 19k). Meanwhile, the covered incident angle range of Gr decreased (Supplementary Figure 19b) and thus the “red” on Gr turned to “yellow” (Supplementary Figure 19k). With the further bending of the middle finger to the palm (Supplementary Figure 19c), the middle finger cannot cover the incident angle range of Gg and thus “green” turned off in this case (p9, Supplementary Figure 19l). The selective interaction between fingers and gratings converted features of gestures into patterns of IR structural colors with minimized interference of self-occlusion. As shown in Supplementary Figure 19j-l, the three IR color patterns can be clearly differentiated based on the color of Gr and Gg. The minimized interference of self-occlusion can enhance the recognition performance of HMI.

Direct gesture-based HMI includes four stages. Except the capture of gestures by IR camera, other three stages, including gesture segmentation from the background, gesture modeling and feature extraction, and gesture recognition as specific commands are all processed by algorithms. The recognition performance is thus highly dependent on algorithms. In comparison, the use of grating array to selectively interact with gestures minimizes the interference of complex background and self-occlusion. The feature of each gesture is directly converted into easily recognizable patterns of IR structural colors. This IR structural color-based HMI thus simplifies the algorithm to directly recognize features of images, with no need of gesture segmentation from the background and the hand modeling and feature extraction. The simplified algorithm is beneficial for real-time HMI without highly required hardware and software.

**Supplementary References**

1. Estakhri, N. M., Neder, V., Knight, M. W., Polman, A. & Alù, A. Visible light, wide-angle graded metasurface for back reflection. *ACS Photonics* **4**, 228-235 (2017).

2. Vollmer, M. & Mollmann, K. P. *Infrared thermal imaging: Fundamentals, research and applications* (Wiley-VCH, 2010).

3. Abutaleb, A. S. Automatic thresholding of gray-level pictures using two-dimensional entropy. *Comput. Vis. Graph. Image Process.* **47**, 22-32 (1989).

4. Cover, T. M. & Thomas, J. A. *Elements of information theory* (John Wiley & Sons, 2001).

5. Wang, M. *et al.* Gesture recognition using a bioinspired learning architecture that integrates visual data with somatosensory data from stretchable sensors. *Nat. Electron.* **3**, 563-570 (2020).

6. Chakraborty, B. K., Sarma, D., Bhuyan, M. K. & MacDorman, K. F. Review of constraints on vision-based gesture recognition for human–computer interaction. *IET Comput. Vis.* **12**, 3-15 (2018).

7. Ge, L., Liang, H., Yuan, J. & Thalmann, D. Real-time 3D hand pose estimation with 3D convolutional neural networks. *IEEE Trans. Pattern Anal. Mach. Intell.* **41**, 956-970 (2019).
