# Supplementary material for: Noncontact human-machine interaction based on hand-responsive infrared structural color
Source: Nat Commun. 2022 Mar 18;13:1446. doi: 10.1038/s41467-022-29197-5 (PMC8933461; doi:10.1038/s41467-022-29197-5)
Supplement: Supplementary file 3 — Description of Additional Supplementary Files [file 41467_2022_29197_MOESM3_ESM.pdf]

Description of Additional Supplementary Files

File name: Supplementary Movie 1

Description: Human-machine interaction based on finger-responsive IR structural colors.

File name: Supplementary Movie 2

Description: Human-machine interaction in the dark.

File name: Supplementary Movie 3

Description: Integration of grating arrays on a wrist band and operation in the dark.
